# Enzymatic Synthesis of 2-Keto-3-Deoxy-6-Phosphogluconate by the 6-Phosphogluconate-Dehydratase From *Caulobacter crescentus*

**DOI:** 10.3389/fbioe.2020.00185

**Published:** 2020-03-20

**Authors:** Sabine Krevet, Lu Shen, Timon Bohnen, Bernhard Schoenenberger, Roland Meier, Markus Obkircher, Klara Bangert, Rudolf Koehling, Eric Allenspach, Roland Wohlgemuth, Bettina Siebers, Christopher Bräsen

**Affiliations:** ^1^Molecular Enzyme Technology and Biochemistry, Environmental Microbiology and Biotechnology (EMB), Centre for Water and Environmental Research, Faculty of Chemistry, University of Duisburg-Essen, Essen, Germany; ^2^Member of Merck Group, Sigma-Aldrich Production GmbH, Buchs, Switzerland; ^3^Institute of Molecular and Industrial Biotechnology, Technical University Lodz, Lodz, Poland

**Keywords:** 2-keto-3-deoxy-6-phosphogluconate, 6-phosphogluconate, 6-phosphogluconate dehydratase, biocatalytic dehydration, *Caulobacter crescentus*, Entner-Doudoroff pathway, metabolite

## Abstract

The availability of metabolic intermediates is a prerequisite in many fields ranging from basic research, to biotechnological and biomedical applications as well as diagnostics. 2-keto-3-deoxy-6-phosphogluconate (KDPG) is the key intermediate of the Entner-Doudoroff (ED) pathway for sugar degradation and of sugar acid and sugar polymer breakdown in many organisms including human and plant pathogens. However, so far KDPG is hardly available due to missing efficient synthesis routes. We here report the efficient biocatalytic KDPG production through enzymatic dehydration of 6-phosphogluconate (6PG) up to gram scale using the 6PG dehydratase/Entner-Doudoroff dehydratase (EDD) from *Caulobacter crescentus* (*Cc*EDD). The enzyme was recombinantly produced in *Escherichia coli*, purified to apparent homogeneity in a simple one-step procedure using nickel ion affinity chromatography, and characterized with respect to molecular and kinetic properties. The homodimeric *Cc*EDD catalyzed the irreversible 6PG dehydration to KDPG with a *V*_max_ of 61.6 U mg^–1^ and a *K*_*M*_ of 0.3 mM for 6PG. Most importantly, the *Cc*EDD showed sufficient long-term stability and activity to provide the enzyme in amounts and purity required for the efficient downstream synthesis of KDPG. *Cc*EDD completely converted 1 g 6PG and a straight forward purification method yielded 0.81 g of stereochemically pure KDPG corresponding to a final yield of 90% as shown by HPLC-MS and NMR analyses.

## Introduction

In the last years high-throughput technologies such as genomics, transcriptomics, proteomics and finally metabolomics have gained increasing importance and have been widely applied in all life science fields e.g., biology, medicine, biotechnology. However, despite this tremendous significance especially the field of metabolomics lacks behind due to the complexity of compounds and matrices but also due to missing standards. Moreover, for classical biochemistry, e.g., enzyme and metabolic pathway characterization, many substrates are not available (Wohlgemuth, 2018).

2-keto-3-deoxy-6-phosphogluconate (KDPG) is the key metabolite of the Entner-Doudoroff (ED) pathway – also known as the KDPG pathway – which is estimated to be utilized by 27% of the heterotrophic prokaryotic microorganisms for sugar and sugar acid (e.g., gluconate) degradation and has recently been found to play also a significant role in cyanobacteria, algae and even higher plants (Flamholz et al., 2013; Chen et al., 2016). Among the ED pathway utilizing organisms there are numerous human pathogens like *Escherichia coli*, *Salmonella enterica*, *Neisseria gonorrhoeae*, *Klebsiella pneumoniae*, *Helicobacter pylori*, *Pseudomonas aeruginosa*, *Legionella pneumophila*, *Campylobacter* spp., and *Pasteurella pestis* (Patra et al., 2012; Vegge et al., 2016; Gonzalez-Mula et al., 2019). In addition, several plant pathogens like *Xanthomonas campestris*, *Pectobacterium carotovorum*, *Agrobacterium tumefaciens* and other organisms of agricultural importance, like Rhizobiaceae, as well as some organisms of biotechnological interest like e.g., *Zymomonas mobilis*, *Gluconobacter oxydans*, are ED pathway utilizers (Stowers, 1985; Richhardt et al., 2012; He et al., 2014). Furthermore, KDPG acts as an intermediate in the degradation pathways of uronic acids as well as of myo-inositol and is therefore involved in the breakdown of complex polymers like e.g., pectin, hyaluron, and sphingolipids (Rodionova et al., 2013; Kuivanen et al., 2019). KDPG is formed in the metabolism via two central routes: (i) In the classical ED pathway KDPG formation takes place after sugar phosphorylation and oxidation from the resulting 6-phosphogluconate (6PG) through a dehydration reaction catalyzed by the so-called Entner-Doudoroff dehydratase (EDD, 6PG dehydratase), a key enzyme of the ED pathway. (ii) In uronic acid degradation and the modified semiphosphorylative ED pathway, known from some bacteria, fungi, and archaea, KDPG formation proceeds via the phosphorylation of the unphosphorylated precursor 2-keto-3-deoxygluconate (KDG) involving KDG kinase. KDPG is then converted to pyruvate and glyceraldehyde-3-phosphate by KDPG aldolase (KDPGA), which represents the second key enzyme in the classical ED pathway (Conway, 1992; Flamholz et al., 2013; Bräsen et al., 2014; Kuivanen et al., 2019). Thus, the ED and related pathways are of broad interest and the availability of pathway intermediates particularly of the marker compound KDPG is crucial for biochemical analyses of enzymes and pathways, as standard for metabolomics for identification, as well as for agricultural and biomedical applications and diagnostics.

For KDPG production, so far one chemical synthesis route and few biocatalytic approaches have been described. For chemical synthesis 3-deoxy-gluconate 6-phosphate was used as starting material which is, however, not commonly available. The procedure included many different synthesis steps and hazardous chemicals like vanadium(v)oxide and potassium chlorate, and the final product was obtained in rather low yields 30–40% in 5 days (Trigalo et al., 1975). As a whole-cell biocatalytic approach, a KDPGA deletion mutant of *Cupriavidus necator* (previously named *Hydrogenomonas eutropha*, *Alcaligenes eutrophus*, or *Ralstonia eutropha*) releasing KDPG to the growth medium and a subsequent purification scheme of the product has been developed (Bowien and Schlegel, 1972; Knappmann et al., 1993). However, this approach relied on the complexity of whole-cell biocatalysts including mutant construction, cell cultivation and its optimization, a multistep product purification from the spent medium, and resulted in relatively low purity (82%). Thus, the reduced complexity of an enzymatic approach seems advantageous. Three of such enzymatic approaches have been described. The first was based on the (partially) purified EDD enzyme from *Pseudomonas putida* enabling a product yield of roughly 80% (O’Connell and Meloche, 1982). Although this method would theoretically allow for a 100% conversion from a thermodynamic point of view, the instability of the enzyme presumably caused by oxidative damage of an iron sulfur cluster hampered its efficient utilization (O’Connell and Meloche, 1982). The second enzymatic method of KDPG synthesis involved the KDPG aldolases from *Pseudomonas fluorescens* catalyzing the condensation of the C3 precursors pyruvate and glyceraldehyde-3-phosphate. The KDPGA from *P*. *fluorescens* and also from *E*. *coli* show the required enantioselectivity only producing KDPG and no 2-keto-3-deoxygalactonate (KDPGal) (Paul Meloche and Wood, 1966; Paul Meloche et al., 1966; Cheriyan et al., 2012). Other KDPG aldolases e.g., from *Z*. *mobilis* show less pronounced stereoselectivity (Seo et al., 2018) and are thus not suited for the synthesis of diastereochemically pure product. However, aldolases catalyze a reversible reaction rendering a 100% conversion of substrate to product impossible meaning that the condensation product needs to be separated/purified from residual substrates, i.e., pyruvate and glyceraldehyde-3-phosphate (O’Connell and Meloche, 1982). The third enzymatic approach described by Lamble et al. (2005) and (Ahmed et al., 2005) involved two enzymatic steps each combined with product purification. In the first step KDG is synthesized from D-gluconate by the gluconate dehydratase (GAD). The KDG production has already been optimized for economic feasibility using the GAD from *Thermoproteus tenax* (Matsubara et al., 2014). In the second step the phosphorylation of KDG is carried out using the KDG kinase. However, the involvement of a second enzyme and the requirement for ATP as a coenzyme renders the procedure complicated and needs intensive purification compared with a desireable one-step approach. Thus, so far no convenient, optimized, and cost efficient route for KDPG production is available.

**FIGURE 1:**
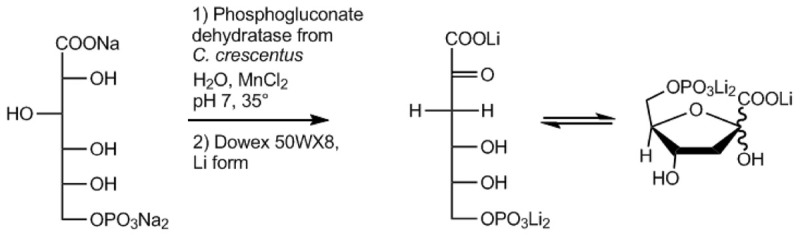
Schematic illustration of the KDPG production scheme developed in this work involving the 6-phosphogluconate dehydratase from *C*. *crescentus*.

Here, we developed a fast, easy, and efficient one-step enzymatic method for the production of KDPG using the EDD from *Caulobacter crescentus* (*Cc*EDD) (Figure 1). *C*. *crescentus* known for its dimorphic life cycle thrives in oligotrophic freshwater habitats and utilizes a variety of carbon sources like carbohydrates, fatty acids, amino acids and aromatic compounds (Poindexter, 2015). Sugars are degraded via the ED pathway involving a functional EDD for KDPG formation (Hottes et al., 2004). The EDD could easily be recombinantly produced from *E*. *coli* using a fast one-step purification. The *Cc*EDD is sufficiently stable and active to produce KDPG with much higher efficiency than in previously described procedures.

## Materials and Methods

### Cloning of the Gene Encoding EDD From *C*. *crescentus* and Functional Overexpression in *E*. *coli*

The EDD encoding gene *CCNA_02134* comprising 1809 bp was amplified from *C*. *crescentus* genomic DNA using the primer set 5′-GTAGATCCATATGAGCCTGAATCCCGTCATC-3′ and 5′-CTAAAGCTTCCTCAAGCAAAGACGGAGGCG3′ (*Nde*I/*Hin*dIII, restriction sites underlined) and cloned into the expression vector pET15b with an N-terminal His-tag. Successful cloning was confirmed by sequencing (LGC genomics, Germany). For expression the resulting construct pET15b-*CCNA_02134* was transformed into *E*. *coli* BL21 (DE3) and cells were grown in 400 ml Luria-Bertani medium supplemented with 100 μg/ml ampicillin and 5 mM manganese chloride. After growth to an OD_600_ of 0.6 at 37°C, expression was induced by adding 0.5 mM IPTG (isopropyl-ß-D-thiogalactopyranoside), and cells were further grown for 17 h at 30°C. Cells were harvested by centrifugation (8,000 × *g*, 15 min, 4°C).

### Purification of EDD From *C*. *crescentus*

The cell pellet was resuspended in buffer A (50 mM NaH_2_PO_4_, 300 mM NaCl, 5 mM MnCl_2_, pH 8) and disrupted by sonication, followed by centrifugation (21,000 × *g*, 45 min, 4°C) to remove cell debris. The supernatant was applied to a Ni-TED (nickel tris-carboxymethyl ethylene diamine) column (MACHEREY-NAGEL GmbH & Co. KG) equilibrated with buffer A. Elution of His-tagged proteins was carried out in elution buffer B (50 mM NaH_2_PO_4_, 300 mM NaCl, 5 mM MnCl_2_, 250 mM Imidazol, pH 8). At this stage the enzyme was essentially pure. Active fractions were pooled, and for long term storage supplemented with 25% glycerol followed by flash freezing in liquid nitrogen, and stored at −80°C. The protein purity was analyzed by sodium dodecyl sulfate-polyacrylamide gel electrophoresis (SDS-PAGE) and the protein concentration was determined using the Bradford assay (QuickStart^TM^, Biorad) with bovine serum albumin as standard. For the determination of the molecular mass under native conditions, pooled EDD samples were concentrated via ultrafiltration (Vivaspin, MWCO 10,000) and applied to a size exclusion chromatography column [HiLoad 26/60 Superdex 200 prep grade column (GE Healthcare)] equilibrated with buffer C (50 mM HEPES/KOH, 300 mM NaCl, 5 mM MnCl, pH 8). Proteins were seperated with an isocratic flow with the same buffer at a flow rate of 2 ml min^–1^.

### Deterimation of the EDD Activity

Activity was determined by coupling the KDPG formation to the oxidation of NADH via KD(P)G aldolase (KD(P)GA) from *Sulfolobus acidocaldarius* (Saci_0225) and L-lactate dehydrogenase (rabbit muscle, Sigma-Aldrich). The KD(P)GA was obtained by PCR amplification of the encoding gene (*saci_0225*) from genomic DNA of *S*. *acidocaldarius* DSM639 using the primers: fd_*Nde*I: 5′-AAACATATGATGGAAATAATTTCACCTATCATTACA, rv_ BamHI: 5′-AAAGGATCCTTAATGTACCAGTTCTTGAATCTT TCT. The restricted PCR product was cloned into pET11c (Novagen). For protein expression and purification conditions refer to Wolterink-van Loo et al. (2007). The KD(P)GA after heat treatment was used as auxiliary enzyme for the characterization of *Cc*EDD. The assay mixtures (0.5 ml) contained 100 mM HEPES buffer pH 8.0, 0.2 mM NADH, 0.675 μg purified *Cc*EDD, 3 U L-lactate dehydrogenase (rabbit muscle, Sigma-Aldrich) and 50 μg *Saci* KD(P)GA-aldolase. After 2 min preincubation at 37°C 6PG was added to start the reaction and the oxidation of NADH to NAD^+^ was followed in a Specord 210 Photometer at 340 nm (Analytik Jena, ε(NADH) = 6.22 mM^–1^cm^–1^). All measurements were performed in triplicates.

### Determination of 6PG Consumption and KDPG Formation in a Discontinuous Assay System

The KDPG formation was assayed in a discontinuous assay (0.5 ml) containing 200 mM HEPES buffer pH 8, 5 mM MnCl_2_, 2.5 mM 6PG and 8.1 μg purified EDD at 37°C. 100 μl samples were removed in regular intervals, and the reaction was stopped by addition of 10 μl 20% (w/v) TCA and incubation on ice for 10 min. After a 15 min centrifugation at 21,000 × *g* and 4°C, 50 μl of the supernatant were mixed with 2 μl 2 M NaOH for neutralization. For KDPG determination 11.4 μl of these samples were added to 100 mM HEPES buffer pH 8, containing 0.2 mM NADH, 3 U L-lactate dehydrogenase (rabbit muscle, Sigma-Aldrich) and 50 μg KD(P)GA-aldolase from *S*. *acidocaldarius* (500 μl final volume) and preincubated at 37°C for 2 min. The oxidation of NADH was followed spectrophotometrically [Analytik Jena, ε(NADH) = 6.22 mM^–1^ cm^–1^]. When the reaction ran to completion 5 μl of the purified EDD (corresponding to 0.22 U and 3.6 μg of protein) was added to determine the residual 6PG. All measurements were performed in triplicates.

### Lab Scale Production of KDPG Lithium Salt

To a solution of 1 g (2.92 mmol) 6PG trisodium salt in 10 ml H_2_O, 390 μl of a solution containing 7 U *Cc*EDD (0.12 mg protein) 50 mM HEPES buffer, 5 mM MnCl_2_, 300 mM NaCl and 25% (v/v) glycerol were added. This mixture, which had a pH of 8.0 was stirred and warmed to 35°C. After 7 h a further 370 μl of the enzyme solution were added and after 3 days thin layer chromatography (TLC) showed complete absence of the starting material 6PG. In process control was done by TLC on silica plates (*n*-PrOH/NH_4_OH (conc.)/H_2_O = 2/1/1, (NH_4_)_2_SO_4_-spray and heat). Additionally, another in process control method was established using HPLC-MS (column: SeQuant ZIC-pHILIC 5 μm, 4,6 × 150 mm, mobile phase A: acetonitrile + 0,1% HOAc, B: 20 mM NH_4_OAc in water, gradient −10 min 25% A, 0.5 min 25% A, 25 min 35% A, 34 min 35% A, flow 1 ml/min, post time 10 min, temp. 45°C, acquisition: microTOF-Q, ESI, negative, 100–650 *m*/*z*, measured values calibrated).

The reaction mixture was cooled to room temperature and filtrated through a centrifugal ultrafiltration unit (MWCO 10,000). To the resulting filtrate (11.2 g) 1.6 g (9.1 mmol) calcium acetate hydrate were added and the pH was adjusted to 4.0 by adding ∼13.6 ml 1 M HCl. To the clear solution 40 ml acetone were added with stirring. The resulting white suspension was cooled in an ice bath, filtrated, the residue washed with 30 ml acetone/H_2_O = 2/1 and then dried on high vacuum to give 0.88 g of a white powder. This was then dissolved in 20 ml H_2_O in an ultrasonic bath and the turbid solution poured onto a column with 40 g of Dowex 50WX8 in its Li^+^ form. Elution with H_2_O, partial concentration on a rotary evaporator at room temperature and lyophilization of the concentrated solution gave 0.81 g (∼90% based on a calculated H_2_O content of ∼10% according to elemental analysis and based on the assumptions that the product is a tri-lithium salt and the starting material had a H_2_O content of 0%) of a pulverizable foam which showed a purity of >97% (TLC) and the expected analytical data as judged by NMR (Supplementary Figures S1–S3) and HPLC–CAD (column: SeQuant ZIC-pHILIC 5 μm 2.1 × 150 mm, mobile phase A: acetonitrile + 0.1% HOAc, Eluent B: 20 mM NH_4_OAc in water, gradient – 10 min 20% A, 0.5 min 20% A, 25 min 35% A, 34 min 35% A, flow 0.3 ml/min, post time 10 min, temperature 40°C, acquisition: HPLC–CAD/MS, ESI, negative mode, CAD: CoronaUltra).

## Results

### Purification and Characterization of the *C*. *crescentus* EDD

The gene *CCNA_02134* encoding the EDD from *C*. *crescentus* was successfully cloned into the pET15b vector and expressed in *E*. *coli* BL21 (DE3) in the soluble fraction. The enzyme was purified to apparent homogeneity in an easy one-step procedure using immobilized metal ion affinity chromatography (NiTED). From 800 ml expression culture corresponding to 4.9 g cell wet weight, 19 mg of apparently pure protein was obtained.

**FIGURE 2:**
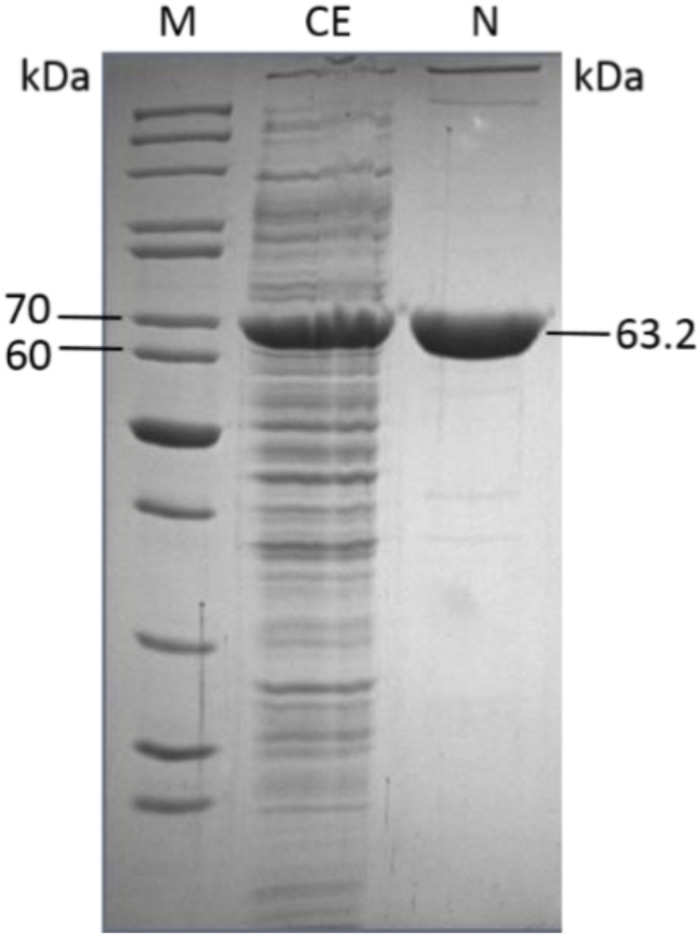
One-step pruification of the recombinant *Caulobacter crescentus* EDD from transformed *E*. *coli* BL21(DE3) as analyzed by SDS-PAGE (M, molecular weight marker PageRuler Thermo Scientific/Fermentas; CE, crude extract; N, purified *Cc*EDD after NiTED).

The molecular weight of the *Cc*EDD was 63.2 kDa under denaturing conditions determined by SDS-PAGE (Figure 2) and 130 kDa under native conditions determined by size exclusion chromatography. This indicates a homodimeric structure of the protein.

**FIGURE 3:**
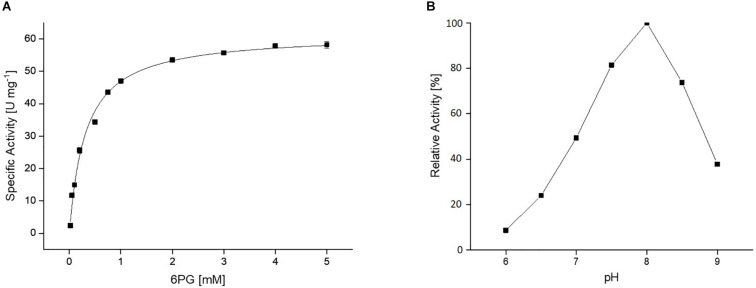
**(A)** Kinetic properties of the recombinant *Cc*EDD. The rate dependence of the *Cc*EDD on 6-phoshogluconate concentration was determined in a continuous assay by coupling the KDPG formation to the NADH oxidation via *S*. *acidocaldarius* KD(P)G aldolase and L-lactate dehydrogenase from rabbit muscle. The activity was determined as absorbance decrease at 340 nm at 37°C. **(B)** Effect of pH on the activity of the *Cc*EDD, 100% activity correspond to a specific activity of 61.6 U mg^– 1^.

The *Cc*EDD catalyzed the dehydration of 6PG to KDPG. The activity was determined in a coupled assay using the KD(P)GA (Saci_0225) and the L-lactate dehydrogenase (rabbit muscle, Sigma-Aldrich) as auxiliary enzymes. The rate dependence of the EDD catalyzed reaction followed classical Michaelis–Menten kinetics with a *V*_max_ of 61.6 U mg^–1^ and a *K*_*M*_ value for 6PG of 0.3 mM (Figure 3A). Dehydratase activity with D-gluconate, D-galactonate, D-xylonate and D-glucose-1-phosphate as substrate was not detected. The pH optimum of the EDD was pH 8.0 (Figure 3B).

**FIGURE 4:**
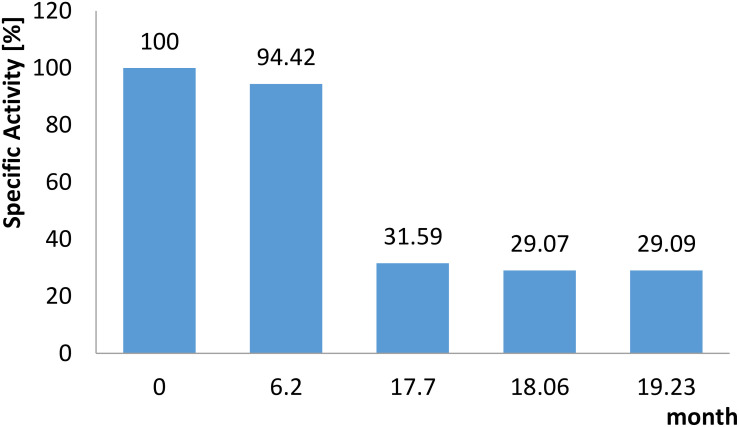
Long term stability of the recombinant *Cc*EDD. The enzyme was stored at –80°C in a buffer composed of HEPES/KOH, pH 8, 25% (v/v) glycerol, 5 mM MnCl_2_. The residual activity was determined at the time points indicated using the continuous assay as described in section “Materials and Methods”.

The addition of 5 mM MnCl_2_ was required during all purification steps and for storage to maintain enzyme activity. In the presence of MnCl_2_ the enzyme lost only 10% activity after 3 days when stored at 4°C. Addition of 25% (v/v) glycerol and storage at −80°C could further stabilize the enzyme: After 30 days 100%, after 6 months 95%, and after 19 months 30% of activity (corresponding to 18 U mg^–1^) was observed (Figure 4). Without addition of glycerol a loss of 30% activity was detected within 30 days.

### KDPG Production

**FIGURE 5:**
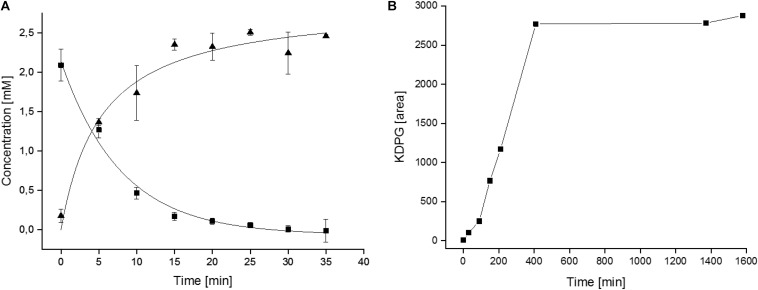
Kinetics of the *Cc*EDD mediated 6PG conversion to KDPG. **(A)** Enzymatic determination of 6PG consumption and KDPG formation during the small scale conversion assays. **(B)** HPLC-MS determination of the KDPG formation (given as increasing peak area in arbitrary units) during lab scale synthesis of KDPG from 6PG.

As a first step toward a larger scale production of KDPG the 6PG conversion to KDPG was analyzed in small scale. Therefore, 2.5 mM 6PG (0.43 mg in 0.5 ml) were incubated in HEPES/KOH, pH 8.0, with 5 mM MnCl_2_ and 8 μg purified EDD (corresponding to 0.16 U) and 6PG consumption and KDPG formation were monitored over time. As indicated in Figure 5A after 20 min the 6PG was completely consumed and the same amount of KDPG was formed indicating a complete conversion of substrate to product.

**FIGURE 6:**
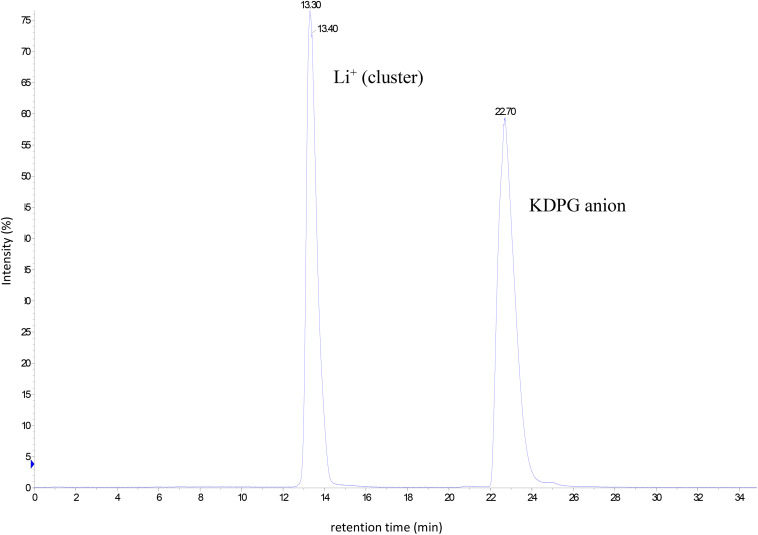
Chromatogram of the HPLC–CAD analysis of the purified product KDPG after lab scale production and purification [0.25 mg ml^–1^, peak at 22.70 min retention time corresponds to a molecular mass of 1 (M–H)– = 258.119 Da, C6H11O9P].

Based on this information the procedure was scaled up to produce KDPG in the lab scale. A solution of 1 g (2.92 mmol) 6PG in a total volume of 10 ml was incubated in the presence of 7 U *Cc*EDD (0.12 mg) as described in section “Materials and Methods”. TLC showed a turnover of ∼30% after 2 h and ∼50% after 5 h. After 7 h further 7 U of the enzyme solution were added to ensure that the enzyme was not limiting. It should be noted that the incubation conditions were different from the small scale conversion (buffer and MnCl_2_ concentrations were 25-fold lower) since the enzyme preparation was added to an aqueous solution of 6PG (finally also to simplify product purification). After 3 days of further incubation TLC showed complete conversion of the starting material (data not shown). However, in another small lot using slightly higher enzyme concentration the conversion was followed by HPLC-MS, which indicated that the formation of KDPG was completed after 400 min (Figure 5B) and thus the changed conditions did not severely impact the short term enzyme stability. The product was analyzed via HPLC-MS/CAD and NMR. Figure 6 shows the HPLC-MS/CAD chromatogram of the purified KDPG with one distinct peak at 22.70 min corresponding to an expected molecular mass of 1 (M–H)– = 258.119 Da, C6H11O9P. The second peak at 13.30 min represents the Li cluster. NMRs showed predominantly the 2 anomeric furanoses in a ratio of ∼4:6 as described (Knappmann et al., 1993; Supplementary Figures S1–S3). ^1^H-NMR (D_2_O, 400 MHz, δ): 4.35 (m, 1H), 4.26 (dt, J = 8.1, 4.2 Hz, 1H), 4.10 (m, 1H), 4.00 (m, 1H), 3.74 (m, twice 2H), 2.46 (dd, J = 14.0, 7.7 Hz, 1H), 2.29 (dd, J = 13.7, 6.8 Hz, 1H), 2.16 (dd, J = 13.6, 6.3 Hz, 1H), 1.89 (dd, J = 14.1, 3.8 Hz, 1H); ^31^P-NMR (D_2_O, 162 MHz, δ): 4.40, 4.32; ^13^C-NMR (D_2_O, 101 MHz, δ): 176.94, 176.23, 103.46, 85.90, 85.81, 85.07, 84.99, 71.56, 64.45, 64.40, 63.62, 43.27, 43.06. TLC (n-PrOH/NH_4_OH/H_2_O = 2/1/1): one spot, r_f_ 0.30; [α]_D_^20^ + 10.8°C (c = 0.1 in H_2_O, after 3 h); Elemental analysis. C(found) 23.30%, H(found) 3.84%; C(calc.) 23.30%, H(calc.) 3.81% (calc. as its tri-lithium salt containing 11% H_2_O). The results indicate that 1 g of 6PG was converted to 0.81 g of diastereochemically pure KDPG corresponding to a yield of 90% (based on calcualted H_2_O content, as described in section “Materials and Methods”).

## Discussion

*Caulobacter crescentus* is a well known ED pathway utilizer, harboring all essential ED genes, which were shown to be upregulated during growth on glucose (Hottes et al., 2004). In contrast, due to a missing phosphofructokinase gene homolog, the EMP pathway seems not to be operative in the catabolic direction. However, despite some data on the glucose-6-phosphate dehydrogenase (Shedlarski, 1974), detailed biochemical characterizations of the ED enzymes from *C*. *crescentus* have so far not been reported, and for the EDDs are generally scarce. Here, the EDD from *C*. *crescentus* shown to be essential for growth on glucose (Hottes et al., 2004) was biochemically characterized and a novel *Cc*EDD based KDPG production scheme was developed.

The *Cc*EDD together with other EDD enzymes belong to the IlvD/EDD family including sugar acid and phospho-sugar acid dehydratases as well as the dihydroxyacid dehydratases known from amino acid synthesis pathways. IlvD/EDD members are mostly homodimers or homotetramers (Rahman et al., 2018) and the 120 kDa homodimeric structure of the *Caulobacter* EDD composed of two 63 kDa subunits has also been reported for the enzyme from *Z*. *mobilis* as well as of *Shewanella oneidensis*. The kinetic constants (*K*_*M*_ 0.3 mM and *V*_max_ 61 U mg^–1^) corresponded to a 30-fold lower catalytic efficiency compared to *Z*. *mobilis* (0.04 mM, 245 U mg^–1^) (Scopes and Griffiths-Smith, 1984) but a 10-fold higher one compared to *P*. *putida* (0.6 mM, 11.4 U mg^–1^) (Meloche and Wood, 1964; Wood, 1971). However, there are only scarce reports on the kinetic and biochemical properties of EDD enzymes and also only one crystal structure (incomplete, without a detailed description published) has been reported from *S*. *oneidensis* (pdb 2GP4) (Rahman et al., 2018), which might presumably be due to the instability of the protein (O’Connell and Meloche, 1982). The *Zymomonas* enzyme lost nearly 80% activity within one day without addition of stabilizing agents (Scopes and Griffiths-Smith, 1984).

This instability appeared strongly influenced by bivalent metal ions as also described for *Pseudomonas* and *Zymomonas* (Wood, 1971; Scopes and Griffiths-Smith, 1984). For the *Cc*EDD, Mn^2+^ ions (5 mM) were required during the whole purification procedure as well as for storage and activity. Without Mn^2+^ only residual activity could be recovered upon purification which got further lost rapidly. However, the presence of Mn^2+^ led to sufficient yields of active enzyme with an enhanced stability over serveral days at 4°C. A similar ion dependence was observed for the EDDs from *Z*. *mobilis* and *P*. *putida* (Wood, 1971; Rodriguez et al., 1996), although the stability of these enzymes was much less pronounced even in the presence of Mn^2+^ ions. The *Cc*EDD remained nearly 100% active when stored in the presence of 5 mM MnCl_2_ at 4°C for 24 h whereas the *Zymomonas* enzyme lost 50% activity under comparable conditions (Scopes and Griffiths-Smith, 1984).

Bivalent metal ions do not only stabilize the EDD enzymes but were also described to be crucial for catalysis. As deduced from crystal structures from homologous sugar acid dehydratases the bivalent metal ions mostly Mg^2+^ stabilize the oxyanion intermediate generated during the catalytic cycle (Rahman et al., 2017, 2018). However, due to missing crystal structures the detailed reaction mechanisms of EDD enzymes remains to be elucidated.

The EDD instability has also been attributed to the presence of a 4Fe-4S cluster as an essential cofactor for catalysis. The presence of 4Fe-4S clusters was indicated by spectroscopic measurements as described for the *Zm*EDD (Rodriguez et al., 1996). In contrast, the closely related sugar acid and dihydroxyacid dehydratases usually contain the more stable 2Fe-2S clusters (Rahman et al., 2018). The 4Fe-4S cluster in EDD enzymes appear to be much more susceptible to oxidative damage under aerobic conditions (Rahman et al., 2018). Especially the *E*. *coli* enzyme was shown to be rapidly inactivated by reactive oxygen species (Gardner and Fridovich, 1991). This instability of the *E*. *coli* enzyme could also be observed in course of this study (data not shown), we cloned and expressed the protein and could confirm the EDD activity. But the activity could not be recovered upon purification, even not under the conditions applied for the *Cc*EDD.

Sequence comparisons between structurally characterized sugar acid dehydratases and EDD enzymes suggested distinct complexation modes of the different FeS clusters, which is reflected by sequence alignments (Rahman et al., 2018). The alignment presented in Supplementary Figure S4 suggests that the *Cc*EDD is more similar to the other EDDs e.g., from *S*. *oneidensis* (58% sequence identity) and *Z*. *mobilis* (55% sequence identity) than to the IlvD dehydratases. Particularly, one of three Cys residues involved in the 2Fe-2S cluster complexation in the *C*. *crescentus* xylonate dehydratase is not conserved in EDDs. Instead, another sequence motif containing a Cys conserved only in EDDs including the *Zm*EDD was identified supporting the presence of an 4Fe-4S cluster in the *Cc*EDD. However, the stability of the *Caulobacter* EDD was shown to be much higher and could even be enhanced by addition of glycerol followed by flash freezing in liquid nitrogen and storage at −80°C. Under these conditions the enzyme was sufficiently stable for several month and even after nearly two years 30% of remaining activity was observed. Thus, the *Cc*EDD overcomes the remarkable instability of EDDs known so far as major drawback for application e.g., for KDPG production. The increased stability of the *Cc*EDD makes it possible to provide the enzyme in amounts (>20 mg of enzyme per 1 l of expression culture) and purity (∼90%) required for the efficient downstream synthesis of KDPG. Also, the activity of 61 U mg^–1^ allows for rapid product formation in the industrial scale range within 7 h, and this production rate might even be increased by higher enzyme amounts.

In general, the biotechnological importance of eliminating reactions including dehydrations has been pointed out (Wohlgemuth, 2018) and the dihydroxyacid dehydratase from the IlvD/EDD family has already been employed in *in vitro* enzyme cascade approaches (Guterl et al., 2012). One major advantage of dehydratases for application is the highly exergonic reaction they catalyze [Δ*G*^0^′ − 43.1 kJ mol^–1^ (Flamholz et al., 2011)] enabling a 100% conversion of substrate to product without requirement of expensive coenzymes, auxiliary reactions etc., as shown in the enzyme kinetics for the 6PG to KDPG conversion (Figure 5). Also, in the up-scaled synthesis procedure 6PG was totally converted to KDPG without any detectable remaining substrate as shown by TLC, HPLC-MS, and NMR. This facilitates straightforward downstream product purification and high yields of 90%, which is also better than previously reported for the *Pseudomonas* enzyme preparation (O’Connell and Meloche, 1982). Furthermore, 6PG is relatively inexpensive and easy to synthesize (Seegmiller and Horecker, 1951). Together with the easy one step enzyme purification and the prolonged enzyme stability the developed process for KDPG production is economically much more feasible. In contrast, although some KDPGAs with pronounced enantioselectivity have been reported, the KDPG aldolase based procedure cannot reach 100% conversion due to the reversibility of the reaction (Δ*G*^0^′ + 15.5 kJ mol^–1^) necessitating a more elaborate product purification scheme, and glyceraldehyde-3-phosphate as precursor is comparably expensive.

From a thermodynamic point of view also the KDPG formation from D-gluconate by GAD and KDGK as described in Ahmed et al. (2005) and Lamble et al. (2005) appears suitable since both enzymes also catalyze strongly exergonic reactions. D-gluconate is a relatively inexpensive precursor and – as mentioned above – an optimized method for the industrial scale production of KDG from D-gluconate has already been described (Matsubara et al., 2014). However, the requirement of two enzymes and ATP as co-substrate makes the procedure more laborious and process optimization has so far not been reported, which might also be due to low expression rates for the KDGK employed (Ahmed et al., 2005). However, there is growing interest in such simple but also in more complex enzyme cascades due to several advantages over whole-cell systems (Fessner, 2015). Such a cell-free enzyme cascade approach has been reported for alginate conversion to KDPG and further to GAP and pyruvate (Nishiyama et al., 2017). This is especially appealing for product formation from cheap and more easily available substrates and might also be considered as an alternative for KDPG synthesis. Nevertheless, enzyme cascades are more complex to understand and to optimize them is much more complicated and time consuming (Morgado et al., 2018) than single enzyme systems as reported here.

In summary, the so far available KDPG synthesis procedures were inefficient and economically unfeasible or at least not optimized so far, leading to the unavailability of KDPG for research and application. Herein, we developed for the first time an easy biocatalytic *in vitro* one-step process with a straightforward protein and product purification protocol using the newly characterized *Cc*EDD showing the required stability for in process utilization.

## Data Availability Statement

The gene sequence (CCNA-02134) used for this study can be found at https://www.uniprot.org/uniprot/A0A0H3CB86.

## Author Contributions

SK, LS, TB, KB, EA, RK, and RM performed the experiments. CB, BSi, BSc, MO, and SK wrote the manuscript, which was edited by CB and BSi. CB, RW, and BSi conceived the study. All authors approved the final manuscript.

## Conflict of Interest

BSc, RM, MO, KB, RK, EA, and RW were employed by the company Member of Merck Group, Sigma–Aldrich Production GmbH, Buchs, Switzerland. The remaining authors declare that the research was conducted in the absence of any commercial or financial relationships that could be construed as a potential conflict of interest.

## Supplementary Material

FIGURE S1 ^1^H-NMR spectrum of the isolated KDPG produced by the *Cc*EDD.

FIGURE S2 ^13^C-NMR spectrum of the isolated KDPG produced by the *Cc*EDD.

FIGURE S3 ^31^P-NMR spectrum of the isolated KDPG produced by the *Cc*EDD.

FIGURE S4 Sequence alignment of 6-phosphogluconate dehydratases (EDD), dihydroxy acid dehydratases (DHAD), and xylonate dehydratase (XAD) as well as arabinoate dehydratases (AraD), from the IlvD/EDD superfamily. The alignment shows the strictly conserved sequence residues in black shading and highlights the conservation of the catalytically essential serine (blue) as well as of bivalent metal ion binding residues (red). Also, only two of three cysteins involved in 2Fe-2S complexation in *C*. *crescentus* XAD are conserved throughout the IlvD/EDD family (yellow) and instead of Cys60 in XAD, another Cys residue (Cys114) is conserved in the C-D-G motif (green) in the EDDs. This suggests a different mode of FeS cluster complexation and is an indication for a 4Fe-4S cluster in the EDD enzymes. The alignment was built using ClustalW according to Rahman et al. (2018) by adding the *Cc*EDD analyzed in this work to a subset of the sequences used therein. Gene bank accession numbers: DHAD *E*. *coli* CDZ22543.1; DHAD *S*. *mutans* KZM62800.1; DHAD *C*. *glutamicum* BAV23085.1; DHAD *C*. *acetobutylicum* WP_010966867.1; XAD *C*. *crescentus* ANS60449.1; AraD *R*. *leguminosarum* ANS60454.1; EDD *Z*. *mobilis* WP_013934127.1;EDD *S*. *oneidensis* WP_011072452.1; EDD *C*. *crescentus* NA1000 YP_002517507.1.
