# Corrigendum: Enzymatic Synthesis of 2-Keto-3-Deoxy-6-Phosphogluconate by the 6-Phosphogluconate-Dehydratase From *Caulobacter crescentus*

**DOI:** 10.3389/fbioe.2020.00761

**Published:** 2020-07-28

**Authors:** Sabine Krevet, Lu Shen, Timon Bohnen, Bernhard Schoenenberger, Roland Meier, Markus Obkircher, Klara Bangert, Rudolf Koehling, Eric Allenspach, Roland Wohlgemuth, Bettina Siebers, Christopher Bräsen

**Affiliations:** ^1^Molecular Enzyme Technology and Biochemistry, Environmental Microbiology and Biotechnology (EMB), Centre for Water and Environmental Research, Faculty of Chemistry, University of Duisburg-Essen, Essen, Germany; ^2^Member of Merck Group, Sigma-Aldrich Production GmbH, Buchs, Switzerland; ^3^Institute of Molecular and Industrial Biotechnology, Technical University Lodz, Lodz, Poland

**Keywords:** 2-keto-3-deoxy-6-phosphogluconate, 6-phosphogluconate, 6-phosphogluconate dehydratase, biocatalytic dehydration, *Caulobacter crescentus*, Entner-Doudoroff pathway, metabolite

In the original article, there was an error in the **Funding** statement. The correct number for BMBF e:bio3 initiative HotSySAPP is 031L0078A. The corrected Funding statement appears below:

BSi acknowledges the funding by the Federal Ministry of Education and Research (BMBF). LS and SK received funds within the e:bio3 initiative HotSySAPP (031L0078A). LS acknowledges funding by MERCUR Pr-2013-0010.

Additionally, Roland Wohlgemuth was not included as an author. The corrected **Author Contributions** Statement appears below.

SK, LS, TB, KB, EA, RK, and RM performed the experiments. CB, BSi, BSc, MO, and SK wrote the manuscript, which was edited by CB and BSi. CB, RW, and BSi conceived the study. All authors approved the final manuscript.

The corrected **Conflict of Interest** statement is as follows:

BSc, RM, MO, KB, RK, EA, and RW were employed by the company Member of Merck Group, Sigma-Aldrich Production GmbH, Buchs, Switzerland.

The remaining authors declare that the research was conducted in the absence of any commercial or financial relationships that could be construed as a potential conflict of interest.

The authors apologize for these errors and state that this does not change the scientific conclusions of the article in any way. The original article has been updated.

