# Supplementary material for: Enzymatic Synthesis of 2-Keto-3-Deoxy-6-Phosphogluconate by the 6-Phosphogluconate-Dehydratase From Caulobacter crescentus
Source: Front Bioeng Biotechnol. 2020 Mar 20;8:185. doi: 10.3389/fbioe.2020.00185 (PMC7099567; doi:10.3389/fbioe.2020.00185)
Supplement: FIGURE S1 — 1H-NMR spectrum of the isolated KDPG produced by the CcEDD. [file Data_Sheet_1.pdf]

# Supplementary Material

## Enzymatic synthesis of 2-keto-3-deoxy-6-phosphogluconate by the 6-phosphogluconate-dehydratase from *Caulobacter crescentus*

**Sabine Krevet[1], Lu Shen[1], Timon Bohnen[1], Bernhard Schoenenberger[2], Roland Meier[2], Markus Obkircher[2], Klara Bangert[2], Rudolf Koehling[2], Eric Allenspach[2], Bettina Siebers[1*], and Christopher Bräsen[1*]**

[1]Molecular Enzyme Technology and Biochemistry (MEB), Envrionmental Microbiology and Biotechnology (EMB), Centre for Water and Environmental Research (CWE), Faculty of Chemistry, University of Duisburg-Essen, 45141 Essen, Germany

[2] Member of Merck Group, Sigma-Aldrich Production GmbH, Industriestrasse 25, 9471 Buchs, Switzerland

*** Correspondence:**

Christopher Bräsen, christopher.braesen@uni-due.de
Bettina Siebers, bettina.siebers@uni-due.de

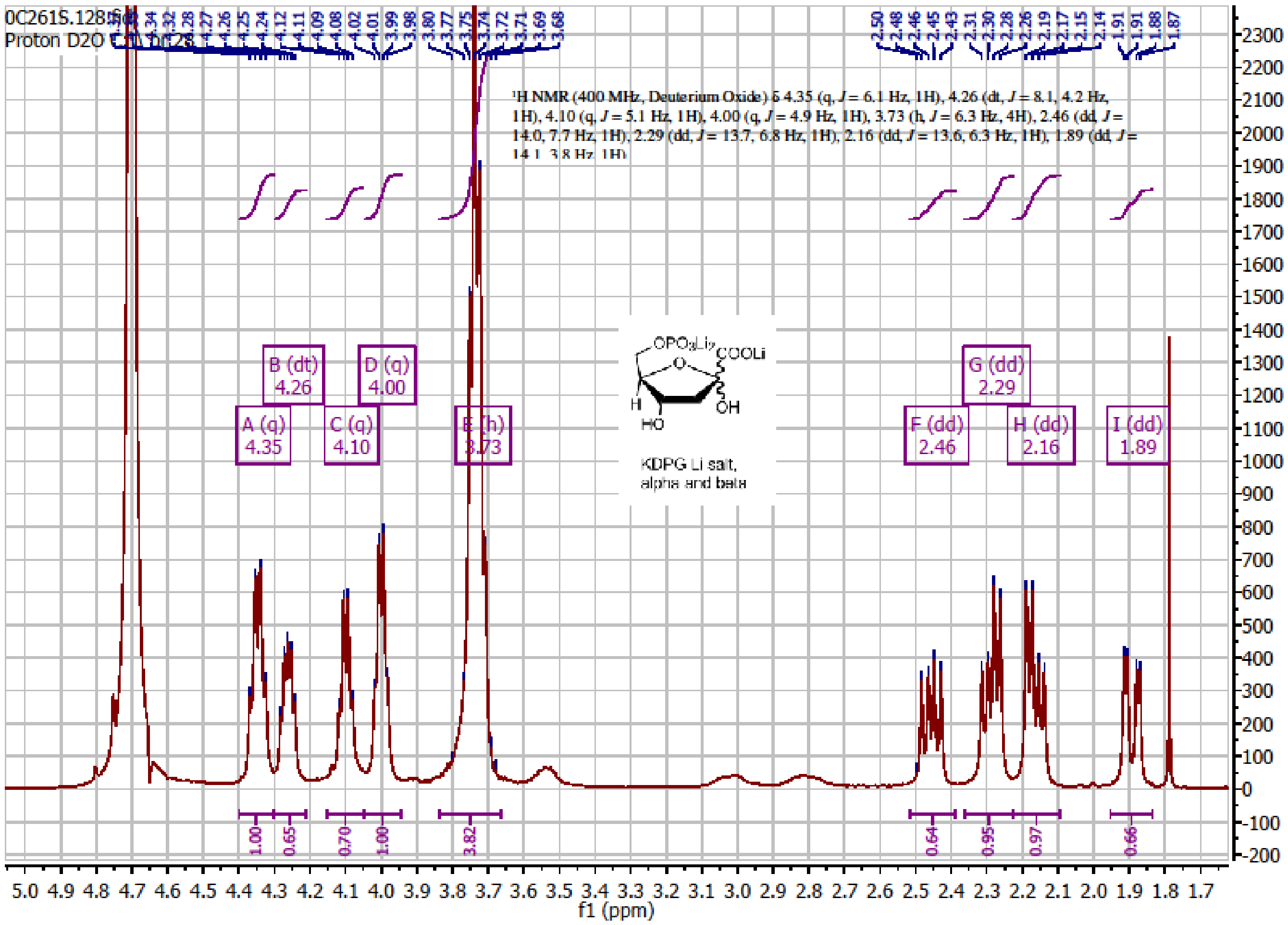


**Supplementary FIGURE 1 |** $^{1}$H-NMR spectrum of the isolated KDPG produced by the *Cc*EDD.

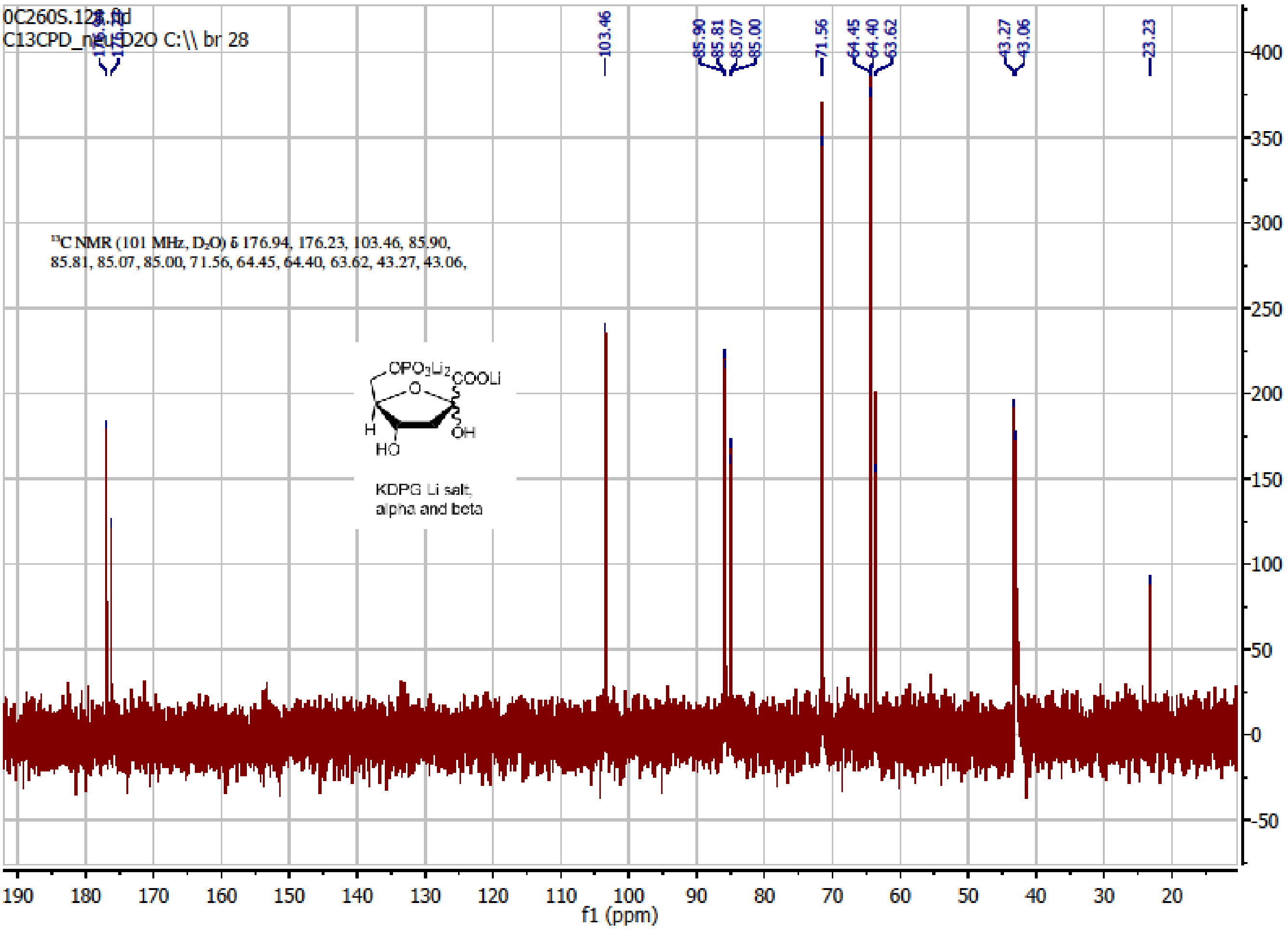


**Supplementary FIGURE 2 |** $^{13}$C-NMR spectrum of the isolated KDPG produced by the *Cc*EDD.

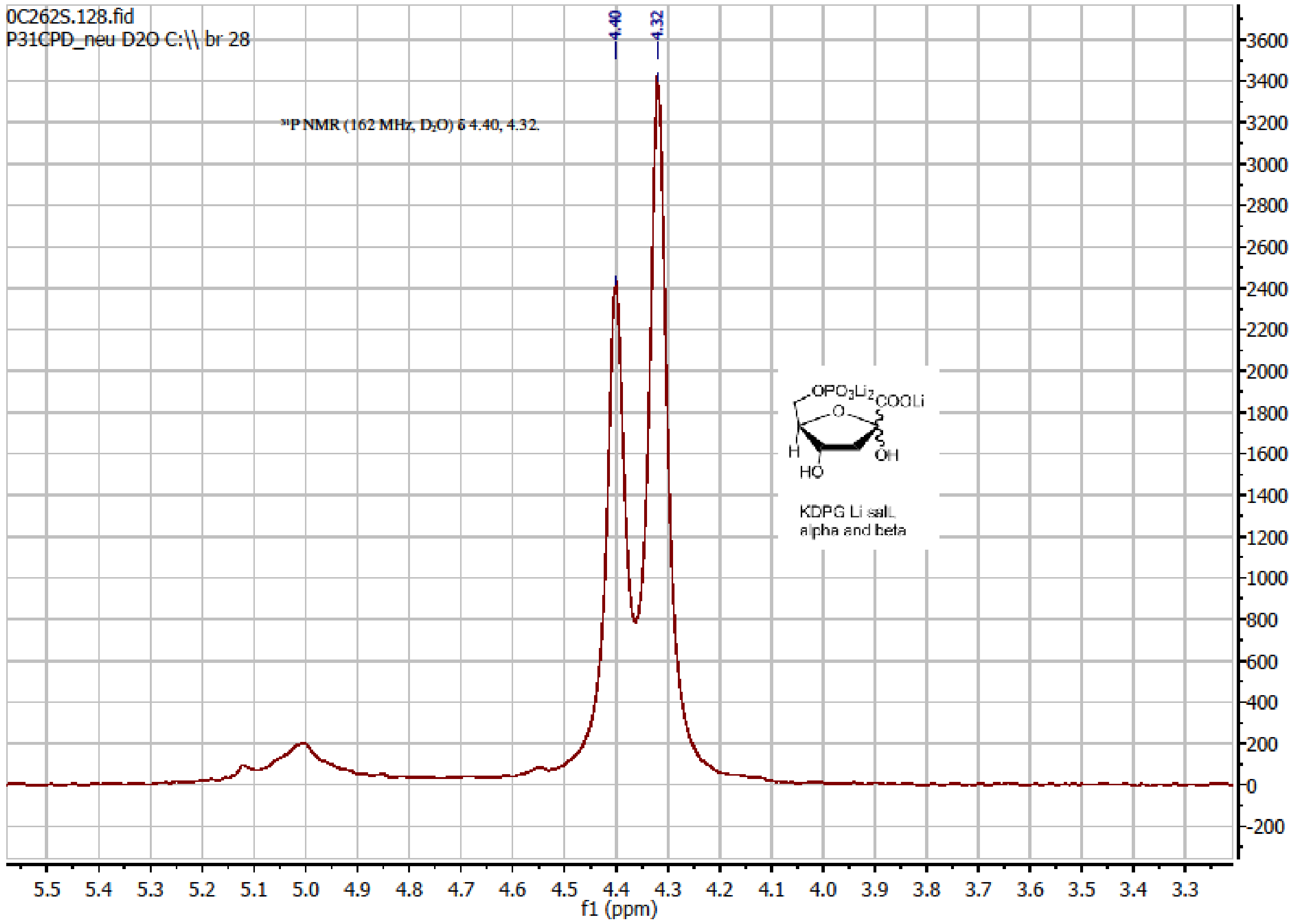


**Supplementary FIGURE 3 |** $^{31}$P-NMR spectrum of the isolated KDPG produced by the *Cc*EDD.

```
DHAD E.coli              -----------------------------MPKYRSATTTHGRNMAG-ARALWRATGMTDA----DFGKPIIAVVNSFTQFVPGHVHLRDL 56
DHAD C.glutamicum        -----------------------------MIPLRSKVTTVGRNAAG-ARALWRATGTKEN----EFGKPIVAIVNSYTQFVPGHVHLKNV 56
DHAD C.acetobutylicum    ----------------------------------MLKSQEIRQKAPELDSLRLGSGWKSE----ELNKAQIIIESSFGHSHPGSAHLDTL 52
DHAD S.mutans            -----------------------MTDKKTLKDLRNRSSVYDSMVKSPNRAMLRATGMQDE----DFEKPIVGVISTWAENTPCNIHLHDF 63
XAD  C.crescentus        --------MDWSHPQFEKSN--RTPRRFRSRDWFDNPDHIDMTALYLERFMNYGITPEEL----RSGKPIIGIAQTGSDISPCNRIHLDL 76
AraD R.leguminosarum     --------MDWSHPQFEKKKKAEWPRKLRSQEWYGG---TSRDVIYHRGWLKNQGYPHDL----FDGRPVIGILNTWSDMTPCNGHLREL 75
EDD  Z.mobilis           MTDLHSTVEKVTARVIERSRKTRKAYLDLIQCEREHGVDRPNLACGNLAHGFAAMG-QDKAALRDFNRMNIGVVTAYNDMLSAHEPYYRY 89
EDD  S.oneidensis       ---MHSVVQSVTDRIIARSKASREAYLAALNDARNHGVHRSSLSCGNLAHGFAACNPDDKNALRQLTKANIGIITAFNDMLSAHQPYETY 87
EDD  C.crescentus        -MSLNPVIADVTARIVARSKDSRAAYLANMDRAIENQPGRAKLSCANWAHAFAASPGVDKLRALDPNAPNIGIVSAYNDMLSAHQPLEAY 89

DHAD E.coli              GKLVAEQIEAAGGVAKEFN-TIAVDDGIAMGHGGMLYSLPSRELIADSVEYMVNAHCADAMVCISNCDKITPGMLMASLRL-NIPVIFVS 144
DHAD C.glutamicum        GDIVADAVRKAGGVPKEFN-TIAVDDGIAMGHGGMLYSLPSREIIADSVEYMVNAHTADAMVCISNCDKITPGMLNAAMRL-NIPVVFVS 144
DHAD C.acetobutylicum    VDEAFKSIDDNGGRGAKYF-VTDICDGETQGHDGMNYSLASRDIMTNLMEIHVQATPFDAGIFIASCDKAVPAHLMAIARL-DMPSILVP 140
DHAD S.mutans            GKLAKVGVKEAGAWPVQFG-TITVSDGIAMGTQGMRFSLTSRDIIADSIEAAMGGHNADAFVAIGGCDKNMPGSVIAMANM-DIPAIFAY 151
XAD  C.crescentus        VQRVRDGIRDAGGIPMEFP-VHPIFENCRR-----PTAALDRNLSYLGLVETLHGYPIDAVVLTTGCDKTTPAGIMAATTV-NIPAIVLS 159
AraD R.leguminosarum     AEKVKAGVWEAGGFPLEVP-VFSASENTFR-----PTAMMYRNLAALAVEEAIRGQPMDGCVLLVGCDKTTPSLLMGAASC-DLPSIVVT 158
EDD  Z.mobilis           PEQMKVFAREVGATVQVAGGVPAMCDGVTQGQPGMEESLFSREVIALATSVSLSHGMFEGAALLGICDKIVPGLLIGALRFGHLATVLVP 179
EDD  S.oneidensis       PDLLKKACQEVGSVAQVAGGVPAMCDGVTQGQPGMELSLLSREVIAMATAVGLSHNMFDGALLLGICDKIVPGLLIGALSFGHLPMLFVP 177
EDD  C.crescentus        PALIKDAARDVGATAQFAGGVPAMCDGVTQGRPGMELSLFSRDVIAMATAVALTHDAFDSALYLGVCDKIVPGLVIGALTFSHLPALFVP 179

DHAD E.coli              GGPMEAGKTKLSDQIIKL--DLVDAMIQGADPKVSDSQSDQVERSACPTCGSCSGMFTANSMNCLTEALGLSQPGNGSLLATHADRKQLF 232
DHAD C.glutamicum        GGPMEAGKAVVVDGVAHAPTDLITAISASASDAVDDAGLAAVEASACPTCGSCSGMFTANSMNCLTEALGLSLPGNGSTLATHAARRALF 234
DHAD C.acetobutylicum    GGIMNAGP------NMLTLEQIGTYNAQYERGEITKEQYEHYKQNACPSCGACSFMGTASTMQVMSEALGIALPGTALIPVTSKELKLAA 224
DHAD S.mutans            GGTIAPGN---LDGKDIDLVSVFEGVGHWNHGDMTKEEVKALECNACPGPGGCGGMYTANTMATAIEVLGLSLPGSSSHPAESAEKKADI 238
XAD  C.crescentus        GGPMLDGWHE--NELVGSGTVIWRSRRKLAAGEITEEEFIDRAASSAPSAGHCNTMGTASTMNAVAEALGLSLTGCAAIPAPYRERGQMA 247
AraD R.leguminosarum     GGPMLNGYFR--GERVGSGTHLWKFSEMVKAGEMTQAEFLEAEASMSRSSGTCNTMGTASTMASMAEALGMALSGNAAIPGVDSRRKVMA 246
EDD  Z.mobilis           SGPMTTGIPN---------KEKIRIRQLYAQGKIGSKELLDMEAACYHAEGTCTFYGTANTNQMMMEVMGLHVPGAAFVTPGTPLRQQLT 260
EDD  S.oneidensis       AGPMKSGIPN---------KEKARIRQQFAQGKVDRAQLLEAEAQSYHSAGTCTFYGTANSNQLMLEVMGLQLPGSSFVNPDDPLREALN 258
EDD  C.crescentus        AGPMTSGLPN---------SEKARIRALYAEGKVGREELLAAESASYHGPGTCTFYGTANTNQMLMELMGFHLPGSAFVHPNTPLREALV 260

DHAD E.coli              LNAGKRIVELTKRYYEQNDESALPRNIASKAAFENAMTLDIAMGGSTNTVLHLLAAAQEAEIDFTMSDIDKLSRKVPQLCKVAPSTQKYH 322
DHAD C.glutamicum        EKAGETVVELCRRYYGEEDESVLPRGIATKKAFENAMALDMAMGGSTNTILHILAAAQEGEVDFDLADIDELSKNVPCLSKVAPNSD-YH 323
DHAD C.acetobutylicum    KNAGKQILKLIELNIK-------PSEIMTKKAFENAIMVHAAIAGSSNCLLHIPAIAHELGMDIEPELFDEIHKKIPYILNIRPSGF-YP 306
DHAD S.mutans            EEAGRAVVKMLEMGLK-------PSDILTREAFEDAITVTMALGGSTNSTLHLLAIAHAANVELTLDDFNTFQEKVPHLADLKPSGQ-YV 320
XAD  C.crescentus        YKTGQRIVDLAYDDVK-------PLDILTKQAFENAIALVAAAGGSTNAQPHIVAMARHAGVEITADDW-RAAYDIPLIVNMQPAGK-YL 328
AraD R.leguminosarum     QLTGRRIVQMVKDDLK-------PSEIMTKQAFENAIRTNAAIGGSTNAVIHLLAIAGRVGIDLSLDDWDRCGRDVPTIVNLMPSGK-YL 328
EDD  Z.mobilis           RAAVHRVTEMGWKGDD----YRPLGKIVDEKSIVNAIVGLLATGGSTNHTLHIPAIARAAGILVNWNDFHDLSEVVPLIARVYPNGP-RD 345
EDD  S.oneidensis       KMAAKQVCRLTELGTQ----YSPIGEVVNEKSIVNGIVALLATGGSTNLTMHIVAAARAAGIIVNWDDFSELSDAVPLLARVYPNGH-AD 343
EDD  C.crescentus        KESARRVAAVTNKGNE----FIPVGRMIDEKSFVNGVVGLMATGGSTNLALHIIAMAAAAGVQLTLEDLDDISKATPLLARVYPNGS-AD 345

DHAD E.coli              MEDVHRAGGVIGILGELDRAGLLNRDVKNVLGLTLPQTLEQYDVMLT-QDDAVKNMFRAGPAGIRTTQAFSQDCRWDTLDDDRANGCIRS 411
DHAD C.glutamicum        MEDVHRAGGIPALLGELNRGGLLNKDVHSVHSNDLEGWLDDWDIRSGKTTEEATELFHAAPGGIRTTEAFSTENRWDELDTDAAKGCIRD 413
DHAD C.acetobutylicum    GSYFWNAGGVPAIMEEIK--EFLHLDVMTVTGKTLGENLEDLKNSGY---------------------YEEHDKLITSLGIKKEDVIRT 372
DHAD S.mutans            FQDLYKVGGVPAVMKYLLKNGFLHGDRITCTGKTVAENLKAFDDLTP-----------------------------------GQKVIMP 374
XAD  C.crescentus        GERFHRAGGAPAVLWELLQQGRLHGDVLTVTGKTMSENLQGRETSDR-------------------------------------EVIFP 380
AraD R.leguminosarum     MEEFFYAGGLPVVLKRLGEAGLLHKDALTVSGETVWDEVKDVVNWNE------------------------------------DVILP 380
EDD  Z.mobilis           VNEFHHAGGMAYVISELLSANLLNRDVMTVSKGGIEDYAKAPVLDDGG----------------------KLVWEPAPKTPGDDTILHP 412
EDD  S.oneidensis       INHFHAAGGMAFLIKELLDAGLLHEDVNTVAGYGLRRYTQEPKLLDG-----------------------ELRWVDGPTVSLDTEVLTS 409
EDD  C.crescentus        VNHFQAAGGMAFVIRELLKAGLVHEDVQTIAGAGLSLYAKEPVLEDG-----------------------MLTWRDGAHESLDPAIVRP 411

DHAD E.coli              LEHAYSKDGGLAVLYGNFAE----NGCIVKTAGVDDSILKF------TGPAKVYESQDDAVEAILGGKVVAGD--VVVIRYEGPKGGPGM 489
DHAD C.glutamicum        VEHAYTADGGLVVLRGNISP----DGAVIKSAGIEEELWNF------TGPARVVESQEEAVSVILTKTIQAGE--VLVVRYEGPSGGPGM 491
DHAD C.acetobutylicum    KENPIQSQGAIAILKGNLAP----GGAVVKHSAISKKLMQV------VLKARVFNCEEDAIKSVLTKNIKPGD--AVFVRYEGPKG-SGM 449
DHAD S.mutans            LENPKREDGPLIILHGNLAP----DGAVAKVSGVK--VRRH------VGPAKVFNSEEEAIEAVLNDDIVDGD--VVVVRFVGPKGGPGM 450
XAD  C.crescentus        YHEPLAEKAGFLVLKGNLFDFAIMKSSVIGEEFRKRYLSQPGQEGVFEARAIVFDGSDDYHKRINDPALEIDERCILVIRGAGPIGWPGS 470
AraD R.leguminosarum     AEKALTSSGGIVVLRGNLAP----KGAVLKPSAASPHLL------VHKGRAVVFEDIDDYKAKINDDNLDIDENCIMVMKNCGPKGYPGM 460
EDD  Z.mobilis           VSAPFSPDGGLRLLEGNLGR------AMYKSSAVDPKFWTI------EAPVQVFSDQDDVQKAFKAGQLDKDV--IVVVRFQGPRA-NGM 487
EDD  S.oneidensis       VATPFQNNGGLKLLKGNLGR------AVIKVSAVQPQHRVV------EAPAVVIDDQNKLDALFKSGALDRDC--VVVVKGQGPKA-NGM 484
EDD  C.crescentus        VSDPFSKEGGLRLMAGNLGR------GVMKISAVKPEHHVI------EAPCAVFQEQEDFIAAFKRGELDRDV--VVVVRFQGPSA-NGM 486

DHAD E.coli              QEMLYPTSFLK-SMGLGKACALITDGRFSGGT-SGLSIGHVSPEAASGGSIGLIEDGDLIAIDIPNRGIQLQVSDAELAARREAQDARGD 577
DHAD C.glutamicum        QEMLHPTAFLK-GSGLGKKCALITDGRFSGGS-SGLSIGHVSPEAAHGGVIGLIENGDIVSIDVHNRKLEVQVSDEELQRRRDAMNAS-E 578
DHAD C.acetobutylicum    PEMFYTTEAIASDHELVDSTALITDGRFSGAT-RGPAIGHVSPEASEGGPIAFVQEGDLIKIDIPARKLDIIGTNGIEKSEKEIENILKE 538
DHAD S.mutans            PEMLSLSSMIV-GKGQGEKVALLTDGRFSGGT-YGLVVGHIAPEAQDGGPIAYLQTGDIVTIDQDTKELHFDISDEELKHRQETIELP-- 536
XAD  C.crescentus        AEVVNMQPPDHLLKKGIMSLPTLGDGRQSGTA-DSPSILNASPESAIGGGLSWLRTGDTIRIDLNTGRCDALVDEATIAARKQDGIPAVP 559
AraD R.leguminosarum     AEVGNMGLPPKVLKKGILDMVRISDARMSGTA-YGTVVLHTSPEAAVGGPLAVVKNGDMIELDVPNRRLHLDISDEELARRLAEWQPNHD 549
EDD  Z.mobilis           PELHKLTPALGVLQDKGYKVALLTDGRMSGATGKVPVALHLSPEALGGGAIGKLRDGDIVRISVEEGKVEALVPAEEWAARPHAEQPA-- 575
EDD  S.oneidensis       PELHKLTPLLGSLQDKGFKVALMTDGRMSGASGKVPAAIHLTPEAIDGGLIAKVQDGDLIRVDALTGELSLLVSDTELATRTATEIDLRH 574
EDD  C.crescentus        PELHNLSPSISVLLDRGHKVALVTDGRMSGASGKTPAAIHVTPEAAKGGPLAYVQDGDVIRVNAETGELKIMVDEATLLARTPANVPA-- 574

DHAD E.coli              KAWTPKNRERQVSF-ALRAYASLATSADKGAVRDKSKLGG---- 616
DHAD C.glutamicum        KPWQPVNRNRVVTK-ALRAYAKMATSADKGAVRQVD-------- 613
DHAD C.acetobutylicum    RSQNWVKPAPRYTKGILGLYTRCASSPMKGGYME---------- 572
DHAD S.mutans            ----PLYSRG-----VLGKYAHIVSSASRGAVTDFWKPEETGKK 571
XAD  C.crescentus        ATMTPWQEIYRAHASQLDTGGVLEFAVKYQDLAAKLPRHNH--- 600
AraD R.leguminosarum     LPTSGYAFLHQQHVEGADTGADLDFLKGCRGNAVGKDSH----- 588
EDD  Z.mobilis           FHPGTGRELFALLRQHAPVAEEGAMAILAAAGL----------- 608
EDD  S.oneidensis       SRYGMGRELFGVLRSNLSSPETGARSTSAIDELY---------- 608
EDD  C.crescentus        SKPGFGRELFGWMRSGVGAADAGASVFA---------------- 602
```

**Supplementary FIGURE 4 |** Sequence alignment of 6-phosphogluconate dehydratases (EDD), dihydroxy acid dehydratases (DHAD), and xylonate dehydratase (XAD) as well as arabinoate dehydratases (AraD), from the IlvD/EDD superfamily. The alignment shows the strictly conserved sequence residues in black shading and highlights the conservation of the catalytically essential serine (blue) as well as of bivalent metal ion binding residues (red). Also, only two of three cysteins involved in 2Fe-2S complexation in *C. crescentus* XAD are conserved throughout the IlvD/EDD family (yellow) and instead of Cys60 in XAD, another Cys residue (Cys114) is conserved in the C-D-G motif (green) in the EDDs. This suggests a different mode of FeS cluster complexation and

is an indication for a 4Fe-4S cluster in the EDD enzymes. The alignment was built using ClustalW according to Rahman et al. (2018) by adding the *Cc*EDD analyzed in this work to a subset of the sequences used therein. Gene bank accession numbers: DHAD *E. coli* CDZ22543.1; DHAD *S. mutans* KZM62800.1; DHAD *C. glutamicum* BAV23085.1; DHAD *C. acetobutylicum* WP_010966867.1; XAD *C. crescentus* ANS60449.1; AraD *R. leguminosarum* ANS60454.1; EDD *Z. mobilis* WP_013934127.1;EDD *S. oneidensis* WP_011072452.1; EDD *C. crescentus* NA1000 YP_002517507.1.
